# The Relationship Between Acute Exercise-Induced Changes in Extramuscular Connective Tissue Thickness and Delayed Onset Muscle Soreness in Healthy Participants: A Randomized Controlled Crossover Trial

**DOI:** 10.1186/s40798-022-00446-7

**Published:** 2022-04-28

**Authors:** Sarah Tenberg, Kazunori Nosaka, Jan Wilke

**Affiliations:** 1 Present Address: Department of Computer Science/Therapy Sciences, University of Applied Sciences Trier, Schneidershof, 54293 Trier, Germany; 2 Centre for Human Performance, School of Medical and Health Sciences, Edith Cowan University, Joondalup, Australia; 3 Institute of Occupational, Social and Environmental Medicine, Goethe University, Frankfurt, Germany; 4 Department of Sports Medicine and Exercise Physiology, Goethe University, Frankfurt, Germany

**Keywords:** Connective tissue, Morphology, Ultrasound, Muscle damage, Biceps brachii, Muscle pain

## Abstract

**Background:**

The extramuscular connective tissue (ECT) has been shown to play a significant role in mechanical force transmission between musculoskeletal structures. Due to this and owing to its tight connection with the underlying muscle, the ECT may be vulnerable to excessive loading. The present study aimed to investigate the effect of eccentric elbow flexor exercise on the morphology of the biceps brachii ECT. In view of the high nociceptive capacity of the ECT, an additional objective was to elucidate the potential relationship between ECT damage and the occurrence of delayed onset muscle soreness (DOMS).

**Methods:**

Eleven healthy participants (♂ = 7; 24 ± 2 years) performed fatiguing dumbbell elbow flexor eccentric exercise (EE) for one arm and concentric exercise (CE) for the other arm in random order and with random arm allocation. Before, immediately after and 24–96 h post-exercise, maximal voluntary isometric contraction torque of the elbow flexors (dynamometer), pressure pain (algometer), palpation pain (100 mm visual analog scale), biceps brachii ECT thickness and ECT/muscle mobility during passive movement (both high-resolution ultrasound) were examined.

**Results:**

Palpation pain, suggestive of DOMS, was greater after EE than CE, and maximal voluntary isometric contraction torque decreased greater after EE than CE (*p* < .05). Relative to CE, EE increased ECT thickness at 48 (+ 17%), 72 (+ 14%) and 96 (+ 15%) hours post-exercise (*p* < .05). At 96 h post-EE, the increase in ECT thickness correlated with palpation pain (*r* = .68; *p* < .05). ECT mobility was not different between conditions, but compared to CE, muscle displacement increased at 24 (+ 31%), 72 (+ 31%) and 96 (+ 41%) hours post-EE (*p* < .05).

**Conclusion:**

Collectively, these results suggest an involvement of the ECT changes in delayed onset muscle soreness.

## Key Points

- Extramuscular connective tissue (ECT) thickness increases after eccentric exercise of the elbow flexors
- Increase in ECT thickness is partly correlated with palpation pain
- Changes in ECT may contribute to the pathogenesis of DOMS; however, evidence of a cause–effect relationship is currently lacking

## Background

The collagenous connective tissue exhibits an intimate mechanical relationship with the skeletal muscle. This is most obvious for the tendon which links muscular and bony structures. However, reducing force transmission to these two tissues is a fallacy [1–4]. Biomechanical experiments have shown that the forces measured at the distal and proximal tendon during muscle lengthening are not equal [1]. It is assumed that the observed proximo-distal force difference stems from mechanical interactions with non-tendinous connective tissues [1]. In some muscles, up to 80% of the fibers do not span the entire distance between origin and insertion [5]. Instead, fusion of adjacent fibers and, with this, an indirect connection between both ends of the muscle are created by the endomysium [5]. It has been demonstrated that mechanical interactions between neighboring muscle fibers occur due to translaminar shearing [6]. Hence, intramuscular force transmission is realized through the fibers themselves and their endomysium. A similar architecture–function relationship can be observed for the perimysium and the deep fascia. Ultrastructural analyses have shown that the perimysium transmits lateral forces between muscle fiber bundles due to direct tissue continuity [7]. The epimysium and the deep fascia, two dense and fibrous layers, encapsulate the skeletal muscle. In some cases, both are not distinguishable from each other [8], and collectively, they form the extramuscular connective tissue (ECT). Interestingly, the ECT interacts with the underlying muscle via fibrous tissue expansions and direct fiber insertions [9, 10]. Recent research has repeatedly investigated the relevance of the ECT for locomotor mechanics. Schleip et al. [11] evaluated the contractility of intrafascial myofibroblasts in response to stimulation with a variety of substances. They concluded that cell-induced alterations of fascial stiffness could be sufficient to impact musculoskeletal dynamics. Wilke et al. [12] used an isokinetic dynamometer to cause a passive movement of the ankle joint. Simultaneously, they measured local movement of the ECT over the Hamstring muscles, which suggests a relevant force transmission via the connective tissue crossing the knee joint. These findings suggest that the ECT seems to be of relevance when examining the flexibility and stability of the musculoskeletal system.

A recent systematic review with meta-analysis has shown that clinically diagnosed muscle injuries rarely affect the muscle tissue in an isolated manner [13]. In contrast, structural lesions mostly occur in the connective tissue, namely the myotendinous junction and the deep fascia. Considering that eccentric overload represents the main mechanism of injury, it has been hypothesized that the connective tissue, via its numerous links to muscular structures, absorbs and dampens excessive elongating forces [13]. Similar to muscle injury, delayed onset muscle soreness (DOMS) is predominantly induced by unaccustomed exercises with eccentric contractions. It could therefore be speculated that the associated tissue overstretch produces micro-lesions in the fascia which contribute to the development of DOMS at 24–72 h post-exercise. This assumption is supported by both histological and experimental studies [14–21]. Due to its dense innervation and equipment with sensory receptors and free nerve endings that act as algogenic nociceptors [14, 15], fascia appears to be of fundamental relevance in pain perception [16–21]. Schilder [20] investigated the pain sensitivity of different structures injecting hypertonic saline into the lumbar fascia and the erector spinae muscle and showed that the irritation of the connective tissue evoked stronger pain responses than the muscle. Gibson [17] performed a similar experiment inducing DOMS by eccentric exercise in one leg. While injection of hypertonic saline into the exercised muscle did not increase pain sensitivity compared to the inactive control leg, a strong increase (exercised vs. control leg) was registered upon irritation of the fascia. Collectively, these findings suggest that the fascia rather than muscle fiber damage seems to cause DOMS. This is supported by data showing that structural muscle damage does not or only weakly correlate with the magnitude of DOMS [22–24].

Although fascia has a high nociceptive potential and may be vulnerable to mechanical overload (e.g., by eccentric contractions), evidence on connective tissue involvement in DOMS is still scare. The aim of the present study, therefore, was to investigate morphological changes in biceps brachii ECT after eccentric exercise of the elbow flexors. Since it is known that concentric contractions do not induce DOMS, morphological and functional changes in the ECT were compared between eccentric and concentric contractions. We hypothesized that, e.g., due to edema and swelling, ECT thickness would increase and ECT mobility would decrease after eccentric exercise only and that the magnitude of the changes in the ECT would be associated with the magnitude of DOMS.

## Methods

### Study Design and Ethics

A randomized, controlled crossover trial with two exercise conditions was performed by 11 young adults. At least 72 h after a familiarization session, each participant visited the laboratory on 5 consecutive days. On day 1, eccentric and concentric exercise (randomized order and arm allocation) of the elbow flexors was performed using a dumbbell with a weight of 80% of the individual one-repetition maximum (1-RM) strength. Outcome measures (see below) were assessed immediately before (base), immediately after (post 0) and every 24 h up to 96 h post-exercise (post 24, 48, 72 and 96). The study was approved by the Edith Cowan University Human Research Ethics Committee and adhered to the Guidelines of Good Clinical Practice as well as the Declaration of Helsinki. All participants provided a written informed consent prior to the study participation.

### Participants

Eleven (seven male and four female) healthy active students (24.3 ± 1.9 years; 167 ± 6 cm; 67.2 ± 8.1 kg; 293 ± 145 min sporting activity per week) volunteered to participate in the present study. The Edinburgh Handedness Inventory—Short Form [25] showed that all participants were right-handed. None of the participants had performed regular upper arm resistance training in the preceding 6 months. All individuals were healthy, not reporting any orthopedic, cardiovascular, neurological, endocrine or psychiatric diseases. Intake of anti-inflammatory or analgesic medicine was prohibited during or on the days before the experiment.

### Determination of One-Repetition Maximum (1-RM)

Immediately before the exercise session, the 1-RM strength in dumbbell curls was assessed (one arm for eccentric exercise and the other arm for concentric exercise) according to the randomization (e.g., left arm eccentric, right arm concentric). This was done because a first bout of eccentric exercise could protect against muscle damage in subsequent bouts of exercise [18, 26]. After a warm-up with a light (50% of the estimated 1-RM) dumbbell (10 elbow flexion and extension movements), the participants performed either one isolated eccentric (lowering the dumbbell) or concentric (lifting the dumbbell) contraction with a load of approximately 80% of the estimated 1-RM. For the 1-RM assessment, each participant sat on a preacher arm-curl bench to stabilize their shoulder*.* For the eccentric 1-RM, the participant was instructed to lower the dumbbell from 90° elbow flexion to full extension in 3 s, and for the concentric 1-RM, the participant lifted the dumbbell from full extension to 90° flexion in 3 s. After each attempt, a 1-min rest was applied, and the procedure was repeated increasing the weight based on the experience in previous studies [27–29] and the perceived effort of the participant until a failed attempt occurred. All participants required less than five attempts for each arm to determine the 1-RM. Calculations of the intra-class correlation coefficient (ICC) and the coefficient of variation (CV) revealed high intraday reliability of this procedure in both males (ICC = 0.993; CV = 0.325 kg) and females (ICC = 0.996; CV = 0.64 kg) [30].

### Exercise Protocols

The participants performed 6 sets of 10 repetitions of eccentric contractions for one arm and concentric contractions of the elbow flexors for the other arm. Repetition duration was 3 s, and the inter-set interval was 2 min. The exercises were performed with a dumbbell, using 80% of the individual 1-RM [31]. After each repetition, the investigator assisted in returning the dumbbell to its starting position to ensure that eccentric-only or concentric-only contractions were performed with the load [32]. If necessary, the investigator carefully supported the participant to complete the protocol. After each set, the participants were asked to rate the effort of the exercise using the rating of perceived exertion (CR-10) scale ranging from 1 anchoring “extremely easy,” to 10 “extremely hard” [33].

### Outcome Measures

The following measurements were taken from the exercised arm before (base), immediately after (excluding muscle pain measures) and 24, 48, 72 and 96 h after exercise. To avoid possible influences of circadian rhythm, examinations were performed at approximately the same time of the day for each participant.

#### Maximal Voluntary Isometric Contraction (MVIC) Torque

**Fig. 1:**
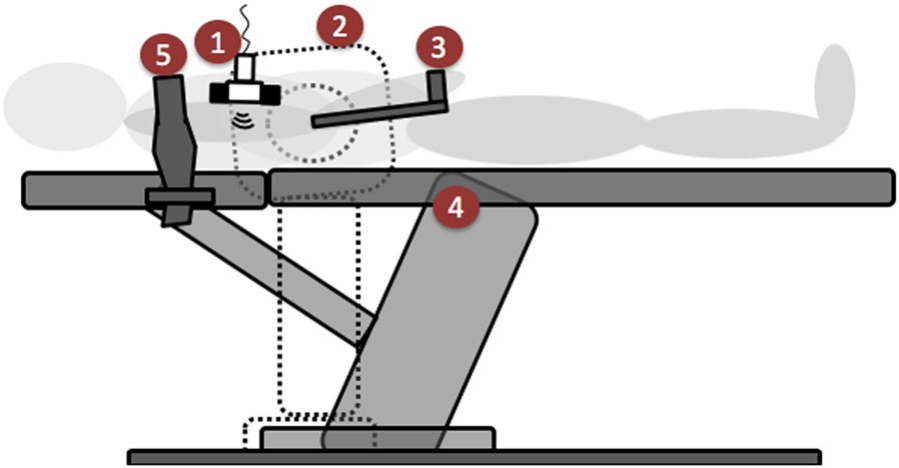
Position of a participant and placement of the isokinetic dynamometer (shown in dashed lines, since it was located behind the therapy table) for the measurement of extramuscular connective tissue (ECT) thickness. (1) Ultrasound transducer, (2) isokinetic dynamometer in 17.5° rotation and 35° elbow flexion, (3) arm bracket of the isokinetic dynamometer, (4) therapy table (5) shoulder stabilization

MVIC torque was measured using an isokinetic dynamometer (Biodex System 3, Biodex medical, Shirley, NY, USA). Data were recorded and stored via the LabChart Pro software (v8.1.5, ADInstruments) to a laptop computer. Each participant was positioned in supine on a therapy bed having the elbow joint aligned with the dynamometer’s axis of rotation (Fig. 1). The elbow joint of the examined arm was positioned at 90° flexion, and both shoulders were stabilized on the therapy bed. Following a brief warm-up, each participant was instructed to flex the elbow joint as fast and strong as possible against the stationary lever arm for 3 s. Verbal encouragement was provided to elicit maximal effort for each attempt. Prieske et al. [34] demonstrated excellent intra- [ICC = 0.99 (0.95–1.00) and CV = 2.0%] and inter-session reliability [ICC = 0.99 (0.96–0.99) and CV = 4.2%] for the used testing approach. Two attempts with a 1-min rest were performed. The average peak force, which could be maintained for 1 s, was considered as the MVIC torque, and the mean of both measurements was used for further analysis [27, 29].

#### Pressure Pain Threshold (PPT)

PPT of the biceps brachii of the exercised arm was measured using an electronic algometer (Somedic AB, Sollentuna, Sweden). Each participant lay on a therapy bed in a relaxed supine position with the forearm being slightly elevated by a soft pad. The probe of the algometer (diameter of 1 cm) was placed on the biceps brachii muscle belly (9 cm above the elbow crease), and compressive force was gradually increased at a rate of approximately 50 kPa/s until the participant reported the first sensation of pain. The value (kPa) corresponding to the force applied at this moment was recorded. After a 10-s rest interval, the procedure was repeated twice, and the average of the two measures was used for further analysis. PPT was measured to indicate pain sensitivity (onset of pain upon pressure). The PPT following eccentric exercise of the biceps brachii muscle, as an average of two measures, has shown a low CV (from 5.6 to 8.9%), small standard errors of measurement (SEM  =  23.3 kPa) and high reliability (ICC from 0.92 to 0.98) [31, 32].

#### Palpation Pain

Muscle pain upon palpation of the elbow flexors was assessed by a 100 mm visual analogue scale (VAS), anchoring 0 being no pain and 100 being the worst possible pain [35, 36]. Each participant was in the same position as that of PPT, and the measurement was taken from the same place as that of PPT (9 cm above the elbow crease). The investigator palpated the muscle of each participant with two fingers in longitudinal orientation [37]. The participants were asked to indicate the level of perceived pain by marking it on the VAS. Pressure was always applied by the same investigator and kept as constant as possible (approximately 400 kPa) between days and participants [27]. The measurement site was marked on the skin with a semipermanent marker and renewed on a daily basis. VAS was measured to indicate the palpation pain intensity (extent of pain induced by the standardized stimulus). VAS measurement upon palpation of the biceps brachii muscle at 9 cm above the elbow crease is highly reliable (ICC from 0.98 to 0.99) and associated with small CVs (from 2.2 to 4.5%) and SEMs (2.6 mm) [31, 32].

#### Extramuscular Connective Tissue Thickness

A high-resolution ultrasound (US) device (Aloka ProSound F75, Hitachi Healthcare, Tokyo, Japan) with an 8.0 × 1.5 cm linear array transducer was used (frequency range of 10 MHz, display depth of 3.0 cm, dynamic range of 60 dB and image gain of 50) to measure ECT thickness. Each participant was positioned in supine with the examined arm being attached to the lever arm of the isokinetic dynamometer at 35° flexion (Fig. 1). The ultrasound probe was positioned longitudinally on the biceps muscle belly (with the center of the probe 9 cm above the elbow crease), double checking the transducer angle to identify beam width artifacts. The investigator ensured to stabilize the probe on the skin with minimal pressure in order to avoid compressing the tissue. Three static images were taken when the deep fascia was clearly recognizable as a hyperechoic region overlying the muscle. All images were immediately checked for artifacts like reverberation or beam width visually, and if artifacts occurred, the images were repeated. To ensure identical positioning during follow-up measurements, the US transducer location was marked on the skin with a semipermanent marker and renewed every day.

**Fig. 2:**
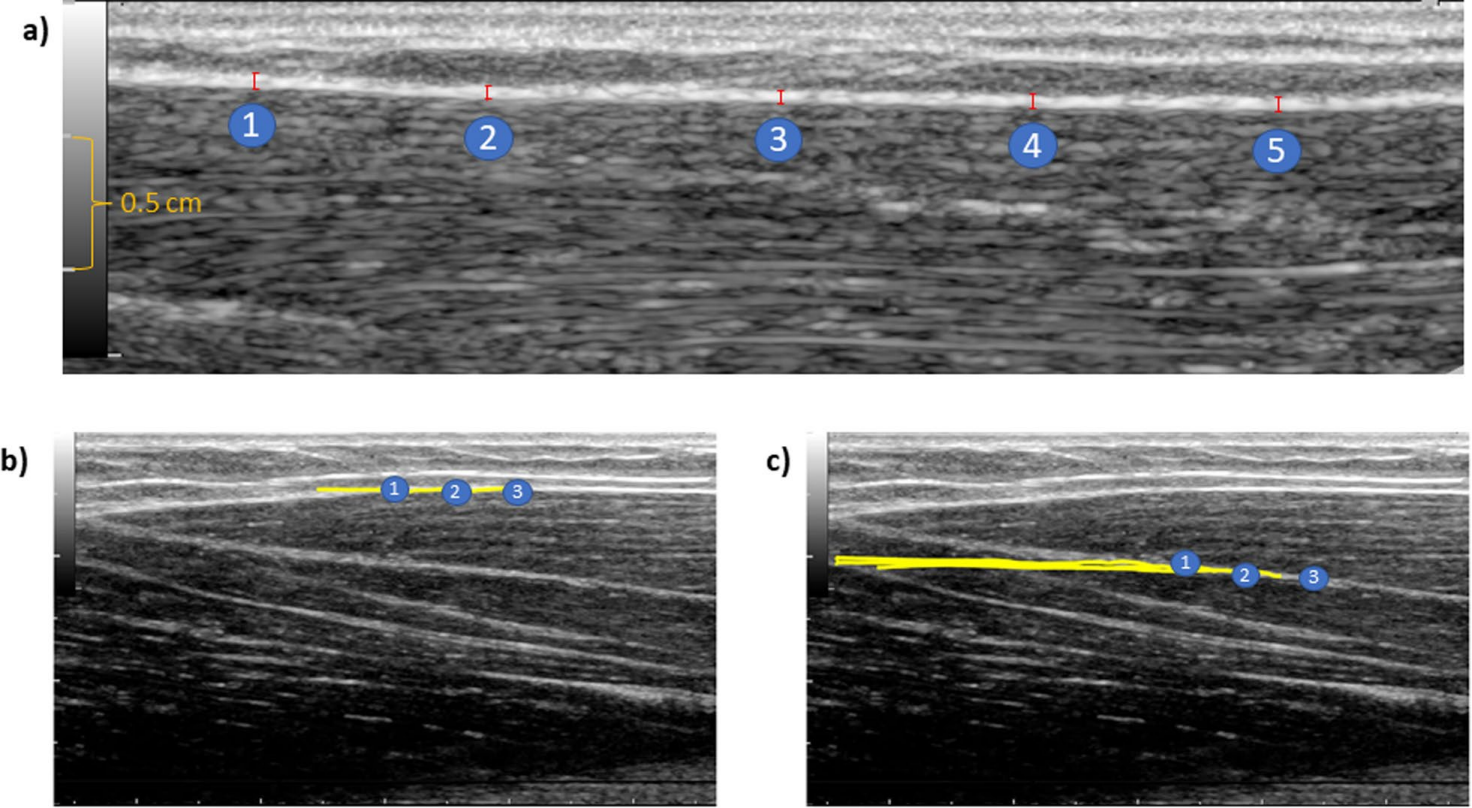
Extramuscular connective tissue (ECT) thickness was measured at five equidistant locations (**a**). The distance between two horizontal lines is 0.5 cm (orange marking). Tissue mobility was measured in the ECT (**b**) and the muscle (**c**) using three regions of interest. Exact indication of horizontal pixel displacement is given in the post-analysis sheet, and accordingly a scale was omitted in the video recording (**b**, **c**)

ECT thickness was calculated using ImageJ (Image J 1.52 k software, USA). Within each US image, five regions of interest (ROIs) at equidistant points were selected for the thickness measurement (Fig. 2a). The average of the five ROIs in each of the three images was chosen to determine the ECT thickness. Thickness measurements via Image J with three averaged images have shown a high inter- and intraday reliability (ICC 0.86–0.98) for determining fascia thickness [38], and the reliability increases with the number of ROIs [39].

#### Extramuscular Connective Tissue and Muscle Displacement

High-resolution ultrasound imaging was also used to evaluate the ECT and muscle displacement during passive movements of the elbow joint of the exercised arm. Each participant was positioned as explained for the MVIC torque measure. The linear array ultrasound transducer was positioned longitudinally on the belly of the biceps brachii with the center of the probe at 9 cm above the elbow crease, and fixed with elastic bandages and tape, ensuring that the muscle was not compressed. The isokinetic dynamometer passively moved the elbow joint three times between 90° flexion and full extension at an angular velocity of 5°/s [40]. Previous studies have shown that reflexive muscle contraction does not occur at this velocity [41, 42]. Participants were instructed to remain completely relaxed, avoiding any voluntary muscle activity. In order to familiarize the participants with the device and measurement conditions, a warm-up of three flexion–extension cycles was performed prior to the actual measurements [12, 43]. Video recordings, depicting ECT and muscle tissue, were made via ultrasound at 10 Hz during the passive movement. Additionally, elbow joint position [°], relative to the neutral zero position, was recorded from the dynamometer signal to the laptop computer. Ultrasound videos were cut into extension and flexion parts of the three repetitions using the synchronized joint position. If a participant could not reach full extension in the follow-up days due to increased muscle stiffness, all videos of this participant were cut to the lowest achieved extension angle. Each video was visually checked for artifacts, which were addressed by removing isolated measurements. If an entire recording of three movement cycles would be characterized by artifacts, the warm-up cycle would be used.

The maximal horizontal displacement of biceps brachii ECT and muscle was quantified using a frame-by-frame cross-correlation algorithm proposed by Dilley [44]. To determine tissue displacement, rectangular ROIs were selected in the ultrasound videos. The used software [44] computes the correlation coefficient between the pixel gray levels of the consecutive frames. The pixel shift with the highest correlation coefficient represents the tissue displacement between the two successive frames. Three equidistant ROIs were defined in the ECT starting 2.5 cm from the myotendinous junction. Additionally, three equidistant ROIs were defined in the muscle on the same level. Horizontal tissue displacement was calculated by calculating the mean of three ROIs and three repetitions for the corresponding tissue (Fig. 2b and c). Averaging multiple ROIs from one repetition has been proven to be a reliable method to measure the displacement of fascia lata (ICC 0.78 to 0.96, SEM 32.6 ± 4.0 mm for the superficial layer, 61.8 ± 5.1 mm for the deep) [43]. A previous study used the mean of three ROIs from one repetition for the calculation of fascial shear strain and demonstrated high intra-rater reliability (0.98) [16].

### Statistical Analyses

All data were tested for normal distribution using a Shapiro–Wilk test, and inference statistics were applied as appropriate. Baseline values were compared between conditions (eccentric vs. concentric) using a paired *t* test or a Wilcoxon test. To examine the possible influence of exercise order (eccentric or concentric arm first), baseline values of participants starting with the respective conditions were compared by means of independent *t* tests or Mann–Whitney U tests. As an additional check, we used point-biserial correlation or contingency tables with Cramer-V to detect potential association of pre–post differences and treatment order.

Prior to the main analysis comparing between eccentric and concentric exercise for changes in the dependent variables over time, sphericity was checked by a Mauchly’s test and the normal distribution of the residuals was examined by a Shapiro–Wilk test. If requirements were met, we would perform two-factorial ANOVAs (condition * time). Resulting effect sizes (*η*^2^) were interpreted as small (0.01), medium (0.06) or large (0.14) according to Cohen [45]. In case of main effects for time, Bonferroni–Holm adjusted post hoc tests (paired *t* test) were performed. Effect size interpretation followed the recommendations of Cohen [45], distinguishing small (*d* =  0.2 to 0.5), medium (0.5 to 0.8) or large (0.8 or higher) effects [45]. In case of violations of the assumptions for parametric testing, Kubinger’s bifactorial rank-variance analyses with Bonferroni–Holm-corrected post hoc Wilcoxon tests were computed. We also investigated possible correlations between ECT thickness, ECT displacement, muscle displacement and palpation pain by a Pearson or Spearman correlation analysis. According to Cohen (1980), correlation coefficients (CC) were interpreted as small (*r* = 0.1 to 0.3), medium (0.3 to 0.5) or large (0.5 and higher) [46]. The main analyses were performed with the pre-post differences.

All analyses were performed with SPSS 26 (SPSS Inc., Chicago, Illinois, USA) and BiAs Statistics (version 11.10, Goethe University, Frankfurt am Main, Germany). The level of significance was set to *α*  =  0.05.

## Results

All eleven participants completed the exercise protocol as planned. However, the muscle/ECT displacement data of one participant had to be excluded due to an erroneous synchronization of the isokinetic testing system and the ultrasound device. Almost no artifacts that would have affected the measurement of fascia thickness were found in the ultrasound recordings, and no videos of the warm-up cycles had to be used. No differences in the baseline values between the two exercise sessions were evident, and no intervention order effects were found (*p* > .05).

### MVIC Torque

**Fig. 3:**
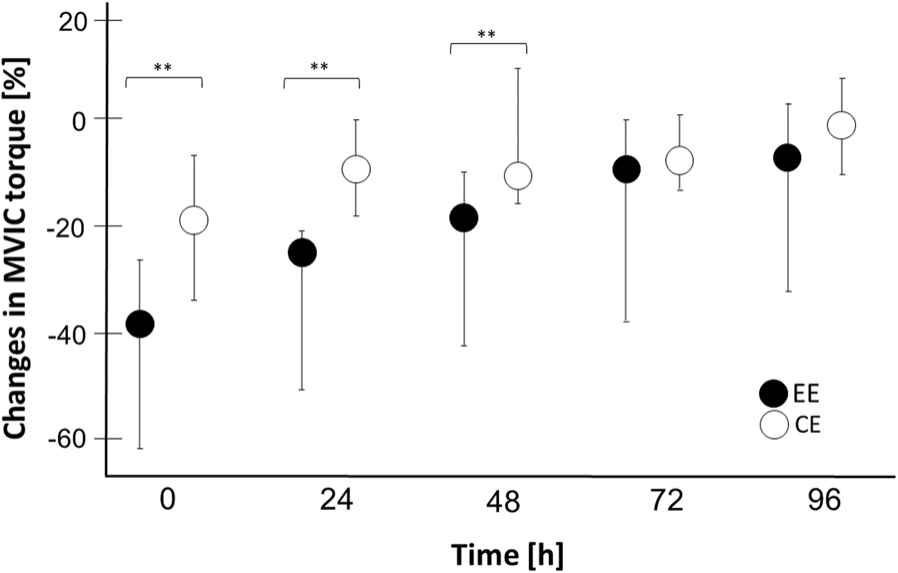
Changes in maximal voluntary isometric contraction (MVIC) torque after exercise. The figure shows nonparametric 95% confidence intervals. Asterisks show significant differences (**for *p* ≤ 0.01) between EE and CE. There were no outliers. EE, eccentric exercise; CE, concentric exercise

The 2 × 5 bifactorial-rank-variance analysis for MVIC showed no interaction effect (*p* > 0.05), but significant main effects for condition (*p* < 0.001) and time (*p* < 0.001). The post hoc analysis for condition showed significantly lower values for EE than CE at post-0 (*r* = − 0.80; *p* = 0.008), post-24 (*r* = − 0.80; *p* = 0.008) and post-48 (*r* = − 0.86; *p* = 0.004) even after Bonferroni–Holm correction (post-0 = 0.024; post-24 = 0.032; post-48 = 0.02; Fig. 3). Likewise, also the post hoc analysis for time identified differences at all measurements (*p* < 0.05), even after Bonferroni–Holm correction.

### PPT

**Fig. 4:**
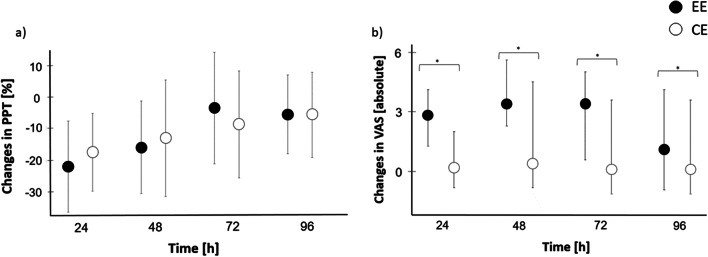
Changes in **a** pressure pain threshold (PPT) and **b** pain on palpation (visual analogue scale, VAS) pain after exercise. The figure shows parametric (PPT) and nonparametric (VAS) 95% confidence intervals. Asterisks show significant differences (*for *p* ≤ 0.05) between EE and CE. There were no outliers. EE, eccentric exercise; CE, concentric exercise

The 2 × 4 ANOVA for PPT did not reveal interactions or main effects for condition (*p* > 0.05), but a main effect for time (*η*^2^ = 0.239; *p* = 0.04). Post hoc testing showed significant differences between post-24 and post-72 (d*z* = − 0.47; *p* = 0.04), post-24 and post-96 (d*z* = − 0.62; *p* = 0.009) as well as post-48 and post-96 (d*z* = − 0.49; *p* = 0.03). However, after Bonferroni–Holm correction, these differences were insignificant (Fig. 4).

### Palpation Pain

The 2 × 4 bifactorial-rank-variance analysis for pain upon palpation revealed no interaction effect (*p* > 0.05). However, a significant main effect was found for time (*p* = 0.01) and condition (*p* < 0.001, Fig. 4). Post hoc tests showed a significant difference between EE and CE for all time points [post-24 (*r* = − 0.89; *p* = 0.003), post-48 (*r* = − 0.75; *p* = 0.013), post-72 (*r* = − 0.70; *p* = 0.021) and post-96 (*r* = − 0.69; *p* = 0.022)], even after the Bonferroni–Holm correction [post-24 (*p* = 0.012), post-48 (*p* = 0.039), post-72 (*p* = 0.042) and post-96 (*p* = 0.022)]. Collectively, these data suggest that EE consistently induced stronger pain (Fig. 4).

### Extramuscular Connective Tissue Thickness

**Fig. 5:**
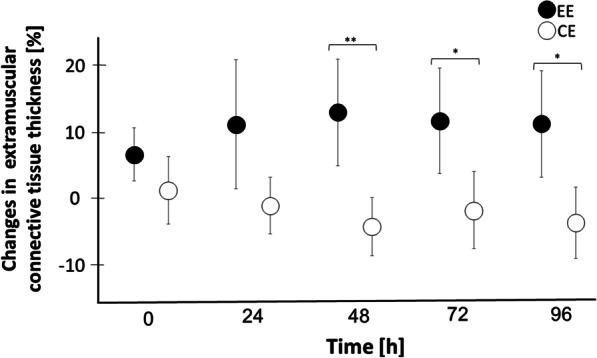
Changes in extramuscular connective tissue (ECT) thickness after exercise. The figure shows parametric 95% confidence intervals. Asterisks show significant differences (*for *p* ≤ 0.05; **for *p* ≤ 0.01) between EE and CE. There were no outliers. EE, eccentric exercise; CE, concentric exercise

The 2 × 5 ANOVA for ECT thickness revealed an interaction effect of time and condition (*η*^2^ = 0.323; *p* = 0.003). The post hoc tests showed differences between EE and CE at post-24 (d*z* = 0.74; *p* = 0.034), post-48 (d*z* = 1.32; *p* = 0.001), post-72 (d*z* = 0.86; *p* = 0.016) and post-96 (d*z* = 0.96; *p* = 0.010). After Bonferroni–Holm correction, only the differences at post-48 (d*z* = 1.32; *p* = 0.005), post-72 (d*z* = 0.86; *p* = 0.048) and post-96 (d*z* = 0.96; *p* = 0.040) were significant (Fig. 5 and Table 1).

**Table 1:** Values for extramuscular connective tissue (ECT) thickness, ECT displacement and muscle displacement in the corresponding condition at the different times including all participants

	Baseline	post	24 h	48 h	72 h	96 h
*Extramuscular connective tissue thickness [mm] #*						
EE	0.6629 ± 0.0996	0.7028 ± 0.0820 (+ 7%)	0.7272 ± 0.0729 (+ 11%)	0.7415 ± 0.0826 (+ 13%)	0.7307 ± 0.0609 (+ 12%)	0.7296 ± 0.0768 (+ 11%)
CE	0.7003 ± 0.0596	0.7075 ± 0.0667 (+ 1%)	0.6921 ± 0.0646 (− 1%)	0.6703 ± 0.068 (− 4%)	0.6877 ± 0.0796 (− 2%)	0.6742 ± 0.0735 (− 4%)
% Difference EE-CE		+ 6%	+ 12%	+ 17%	+ 14%	+ 15%
*Extramuscular connective tissue displacement [mm] §*						
EE	4.54 (2.61–8.57)	5.09 (0.13–17.90)	3.95 (− 1.10 to 16.60)	3.41 (2.19–18.52)	2.89 (1.29–15.85)	2.81 (1.16–20.5)
CE	6.29 (4.46–10.47)	4.95 (1.26–10.61)	6.42 (− 0.37 to 10.36)	6.38 (0.61–11.08)	5.03 (0.96–10.73)	5.40 (0.25–10.66)
*Muscle displacement [mm] #*						
EE	41.28 ± 7.72	49.50 ± 15.72 (+ 21%)	52.87 ± 9.88 (+ 30%)	52.73 ± 12.33 (+ 38%)	51.72 ± 9.49 (+ 31%)	53.62 ± 12.55 (+ 40%)
CE	54.98 ± 11.25	48.96 ± 9.51 (− 9%)	53.83 ± 11.45 (− 1%)	48.57 ± 9.50 (− 6%)	51.41 ± 9.35 (± 0%)	54.23 ± 10.69 (− 1%)
% Difference EE-CE		+ 30%	+ 31%	+ 44%	+ 31%	+ 41%

Values are given in mm. The percentage indicates the increase/decrease compared to baseline

EE, eccentric exercise; CE, concentric exercise, #, mean ± standard deviation, §, median (minimum − maximum))

### Extramuscular Connective Tissue and Muscle Displacement

**Fig. 6:**
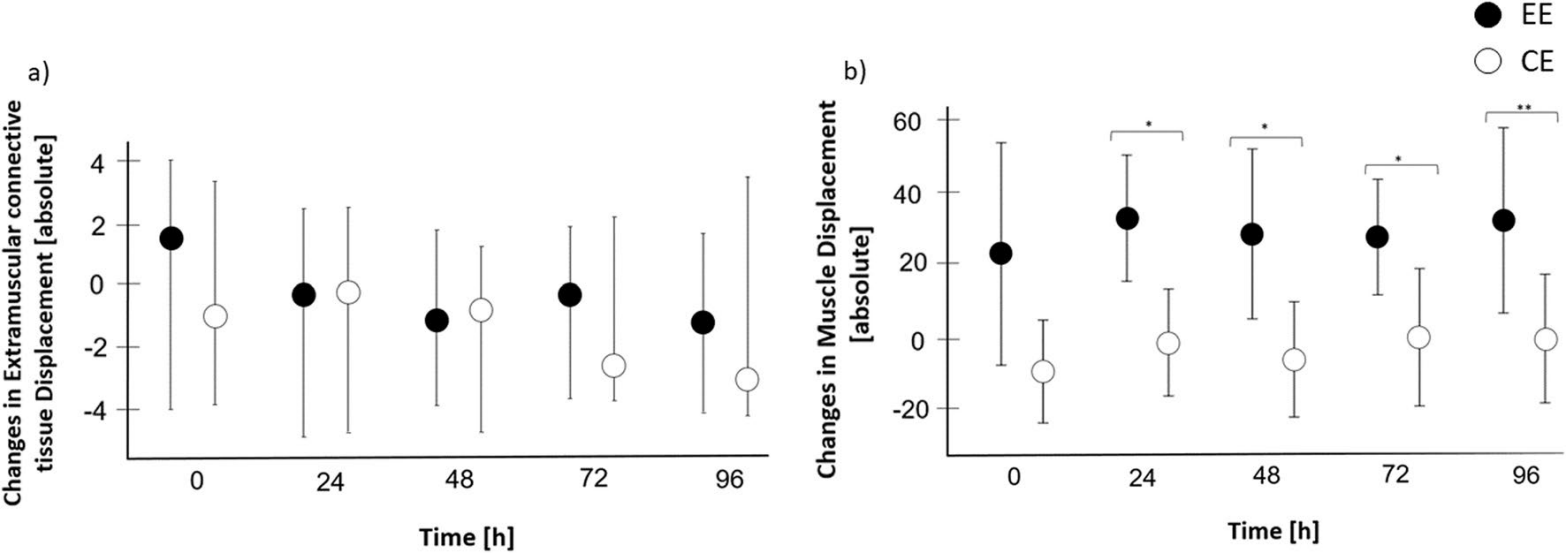
Changes in **a** extramuscular connective tissue (ECT) and **b** muscle displacement after exercise. The figure shows nonparametric (ECT displacement) and parametric (muscle displacement) 95% confidence intervals. Asterisks mark significant differences (*for *p* ≤ 0.05; **for *p* ≤ 0.01). There were no outliers. EE, eccentric exercise; CE, concentric exercise

The 2 × 5 bifactorial rank-variance analysis revealed no interaction effect for either ECT or muscle displacement (*p* > 0.05; Fig. 6, Table 1). However, regarding muscle displacement, a significant main effect for condition was found (*p* = 0.003). Post hoc tests showed a significant difference between EE and CE at post-24 (*T* = − 3.582; *p* = 0.006), post-48 (*T* = − 3.398; *p* = 0.009), post-72 (*T* = − 2.984; *p* = 0.018) and post-96 (*T* = − 4.495; *p* = 0.001). After Bonferroni–Holm correction, most comparisons remained significant (post-24: *p* = 0.024, post-48: *p* = 0.027, post-72: *p* = 0.036, post-96: *p* = 0.005) although one (post-0: *p* = 0.051) failed to meet the significance threshold.

### Correlations

Spearman’s rank correlation showed no significant association of pre–post differences in VAS and ECT thickness for CE. Contrarily, increased ECT thickness following EE correlated strongly with palpation pain at 96 h post-exercise (*r* = 0.682, *p* = 0.021). Additionally, at 72 h post-exercise, there was a tendency for a correlation (*p* = 0.051) with a high positive correlation coefficient (*r* = 0.60).

Regarding tissue displacement measures, only muscle displacement correlated with VAS at post-24 (*r* = − 0.675; *p* = 0.032) and post-96 (*r* = − 0.685; *p* = .029) after CE. Yet, there was a tendency for an association of muscle displacement and VAS pain at post-48 after EE (*r* = 0.626; *p* = 0.053).

## Discussion

Skeletal muscle fiber damage and inflammation have often been discussed as primary sources of DOMS [47–50]. However, the pain occurring after eccentric exercise is not or only weakly related to the structural muscle fiber damage identified in related experiments [22–24]. The present study demonstrated that eccentric exercise increased ECT thickness to a considerable degree, and this increase correlated with participant’s pain sensations. Taken together, our findings suggest a relevant contribution of the ECT to the pathogenesis of DOMS, although a cause–effect relationship is yet to be established.

Changes in pain sensation after eccentric exercise were already evaluated and shown to be related to the fascia in two previous studies [17, 18]. Injection of hypertonic sodium solution and electrical stimulation of the connective tissue caused higher intensities and durations of pain than the irritation of the underlying muscle itself. However, the mechanisms underlying these observations have not been scrutinized. Solomonow [51] proposed a model describing the degradation of viscoelastic tissue after prolonged high-load exercise. Briefly, it suggests that repeated mechanical strain leads to micro-damage to collagen fibers, which, in turn, can trigger inflammation. Indirect signs of collagen breakdown after eccentric exercise have been observed in the form of increased total serum collagen [52, 53] and type IV collagen concentration [54]. Although a cause–effect relationship cannot be derived from our data, it is assumed that the increases in ECT thickness reflect tissue inflammation and/or edema triggered by collagenous micro-failure of the muscular connective tissue, which would be in line with data describing the vulnerability of fascia in muscle injury [16]. We found a nonsignificant decrease in ECT thickness following concentric contractions. From a theoretical point of view, it could be explained by a removal of water from the ECT which has already been observed following stretching [55]. Another explanation could be a change in the mechanical properties of the hyaluronic acid, which is sensitive to movement stimuli [56]. However, as said decrease was not significant, both remains speculative.

We observed an increase in passive muscle tissue displacement after eccentric exercise, but no such change was found in the ECT. Previous studies have revealed that the sliding of the deep fascia against the muscle is a significant functional soft tissue property, which, if altered, may induce muscle pain [16]. We expected that damage of the ECT following eccentric exercise might negatively affect both total fascial mobility and tissue gliding. While the former could not be affirmed by our data, muscle displacement increased after eccentric exercise but not after concentric exercise. This was in line with the findings from Lau et al. [28], who found a significant increase (50%) in biceps brachii myotendinous junction displacement during eccentric elbow flexor contractions from the first to the tenth set in the first bout. After a second bout of the same exercise performed 4 weeks later, no changes in the displacement were evident. Since DOMS typically occurs after the first bout of eccentric exercise but not after the second bout, the authors concluded that the change in muscle length affected the magnitude of muscle damage. Since no intramuscular measurement was performed, the cause of increased muscle movement following eccentric exercise is unclear. However, eccentric contractions involve a stretching stimulus on the muscle and a temporary decrease in muscle stiffness [57] could explain the observation.

With regard to our data, taking together both, the increased muscle displacement in the passive movement after eccentric exercise and the constant ECT mobility suggest that the ECT was unable to adapt the higher muscle movement. Yet, as this observation was not correlated with the magnitude of DOMS, future research is warranted to conclusively judge the relevance of an altered muscle–fascia mobility ratio.

Our findings may have direct implications for exercise professionals designing programs for recreational and elite athletes, particularly in competitive sports, where rapid recovery and restoration of performance are key factors for success. In view of the increasing evidence suggesting an involvement of the ECT in DOMS, treatments providing tissue-specific stimuli could accelerate recovery. Recently, Clifford et al. [58] found a moderate-magnitude reduction in DOMS after supplementation with collagen peptides which would fit with an assumed existence of micro-damage. A plethora of studies, moreover, demonstrated that massage [59–61] and foam rolling [62–65] alleviated DOMS. Finally, it has been repeatedly shown that the ECT generally adapts to mechanical loading [43, 66]. Future research elucidating exercise paradigms addressing the ECT could therefore open new frontiers in the prevention and therapy of DOMS.

Some limitations need to be discussed. Although our 10 MHz ultrasound transducer allowed a detailed visualization of the ECT, a transducer with an even higher frequency (e.g., 15 MHz) may have increased image quality and precision due to larger spatial resolution. Also, to avoid a bias due to variation, we kept the ultrasound settings constant. However, this did not take into account the differences in participants’ anatomy. Adjusting the settings individually could hence have slightly improved the precision of the results. During measurements, no electromyographic recordings were made in order to verify that the movement induced by the isokinetic dynamometer was purely passive. However, this can be assumed with a relatively high degree of certainty as previous studies showed that such continuous passive motion does not provoke muscle activity [41, 42]. Another issue relates to the sample size. Our study was explorative in nature, and the identification of between-condition effects with only 11 (10 for tissue displacement) participants suggests a relevant implication of ECT morphology in DOMS. However, with regard to insignificant parameters (i.e., ECT mobility), future studies with larger sample sizes may still observe small-magnitude effects. Finally, it is worth mentioning that a washout phase between conditions (concentric and eccentric exercise) was omitted. A carryover effect could thus have influenced the measurements from a theoretical point of view; however, it is unlikely that the results were largely affected by a carryover effect (e.g., contralateral repeated bout effect) to make the comparison between eccentric and concentric exercise conditions unreasonable.

## Conclusions

Eccentric exercise increased ECT thickness, which was associated with the magnitude of DOMS. These findings support and expand the available evidence suggesting an involvement of the extramuscular connective tissue in the pathogenesis of DOMS. Future studies should (1) clarify the potential association of eccentric loading, connective tissue damage, local inflammation and DOMS, and (2) develop and test specific exercise paradigms addressing the ECT for recovery.

## Data Availability

The datasets used and analyzed during the current study are available from the corresponding author on reasonable request.

## Abbreviations

1-RM: One repetition maximum
CC: Correlation coefficient
CE: Concentric exercise
DOMS: Delayed onset muscle soreness
ECT: Extramuscular connective tissue
EE: Eccentric exercise
MVIC: Maximal voluntary isometric contraction
PPT: Pressure pain threshold
ROIs: Regions of interest
US: Ultrasound
VAS: Visual analogue scale
